# Serum tsRNA as a novel molecular diagnostic biomarker for lupus nephritis

**DOI:** 10.1002/ctm2.830

**Published:** 2022-05-20

**Authors:** Xiaoshan Zhang, Ping Yang, Adeel Khan, Dongjie Xu, Shanshan Chen, Junbin Zhai, Bin Zhang, Tao Xiong, Yanbo Wang, Zhiyang Li

**Affiliations:** ^1^ College of Life Science Yangtze University Jingzhou China; ^2^ Department of Clinical Laboratory The Affiliated Drum Tower Hospital of Nanjing University Medical School Nanjing China; ^3^ State Key Laboratory of Bioelectronics School of Biological Science and Medical Engineering National Demonstration Center for Experimental Biomedical Engineering Education Southeast University Nanjing China; ^4^ Department of Rheumatic Immunology The Affiliated Drum Tower Hospital of Nanjing University Medical School Nanjing China; ^5^ State Key Laboratory of Pharmaceutical Biotechnology and Department of Physiology Jiangsu Engineering Research Center for MicroRNA Biology and Biotechnology School of Life Sciences NJU Advanced Institute of Life Sciences (NAILS) Nanjing University Nanjing China

Dear Editor,

Systemic lupus erythematosus (SLE) is an autoimmune illness with a lifelong toll on the body. Meanwhile, approximately 30% of SLE patients develop lupus nephritis (LN).^1^ However, clinical diagnosis of LN still relies on the "combination" of clinical symptoms, such as all proteins in the 24‐hour urine of the patient (24‐hour proteinuria) and anti‐double stranded DNA antibodies (anti‐dsDNA).^2^ tRNA‐derived fragments (tsRNAs) are novel noncoding small RNAs (14∼40 nt in length) formed by the cleavage of mature tRNAs or their precursors under the action of enzymes or stress.^3^ tsRNAs have earlier biogenesis than most RNA and protein synthesis and are abundant in serum (> 50%) than miRNAs.^4^, ^5^ Hence, we researched and identified novel serum tsRNA molecular marker for the diagnosis of LN, with prospects to become noninvasive liquid biopsy tool for LN diagnosis.

The sera of LN patients and healthy controls (HCs) were acquired at “Drum Tower Hospital affiliated with Nanjing University School of medicine” (Table 1). Thoroughly following the 1997 American Society of Rheumatology (ACR) , guidelines, signed consent forms from patients and approval from ethics committee (ID: 2020‐327‐01) were obtained. Serum RNA was extracted with TRIzol and treated with a rtStart™ tRF and tiRNA pretreatment kit by Arraystar Inc., USA, before sequencing. cDNA was constructed prior to quantitative reverse transcription polymerase chain reaction (RT–qPCR).^6^ When mapping the sequencing results, only one mismatch was allowed, and the differentially expressed tsRNAs (fold change > 10, *P* value < .01) in LN were selected for subsequent exploration (Figure 1A; and Table S1). tsRNAs of the tRF‐1 type were more abundant in LN serum (Figure. 1B), which may be associated with systemic damage and preference of splice sites of angiopoietins in lupus patients.^7^ Venn analysis showed that 193 tsRNAs were present simultaneously in both groups (Figure 1C). The length range of tsRNA fragments was enriched at bases 20‐23 nt and 30‐32 nt (Figure 1D). Finally, we cluster analysed the 10 highly and 12 lowly expressed tsRNAs in LN (Figure 1A) and selected the highly expressed tsRNAs for marker screening (Figure 1E; and Tables S2, S3). RT–qPCR confirmed overexpression of tRF‐Ala‐AGC‐2‐M4 and tRF‐Gly‐TCC‐1‐M3 in the LN compared to HC (training set, Figure 1F) using agarose gel electrophoresis and sequencing of T vector clones to confirm the specificity of RT–qPCR (Figure S5). A standard curve of quantitative CT values was transformed for tsRNA concentration (Figure S1).

**TABLE 1:** Statistics of clinical information of specimens

**Characteristics**	**Training set**		**Validation set**	
	**HC**	**LN**	**HC**	**LN**
Cases	24	24	46	73
Sex (female, percentage)	22 (91.67%)	19 (79.17%)	33 (71.74%)	68 (93.15%)
Age (mean ± SD, year)	38.92 ± 2.75	41.75 ± 3.21	32.13 ± 1.11	41.88 ± 1.64
WBC (mean ± SD, 10^∧^9/L)	5.20 ± .34	5.38 ± .51	6.04 ± .26	6.44 ± .49
RBC (mean ± SD, 10^∧^12/L)	4.47 ± .08	3.40 ± .13	4.66 ± .06	3.48 ± .08
HGB (mean ± SD, g/L)	131.96 ± 2.68	103.42 ± 4.51	140.27 ± 2.36	100.16 ± 2.85
HCT (mean ± SD, %)	39.57 ± .76	30.48 ± 1.21	41.63 ± .60	30.59 ± .74
PLT (mean ± SD, 10^∧^9/L)	256.96 ± 10.72	161.29 ± 18.56	254.55 ± 10.18	172.35 ± 11.07
Ne (mean ± SD, 10^∧^9/L)	3.03 ± .26	3.91 ± .39	3.64 ± .20	5.10 ± .44
Ly (mean ± SD, 10^∧^9/L)	1.75 ± .09	1.00 ± .14	1.90 ± .08	.92 ± .06
Mo (mean ± SD, 10^∧^9/L)	.28 ± .02	.41 ± .05	.33 ± .02	.39 ± .04
eGFR (mean ± SD)	127.77 ± 5.11	117.95 ± 11.70	132.99 ± 3.40	110.73 ± 6.68
Anti‐dsDNA (mean ± SD, IU/L)	N/A	297.91 ± 88.60	.91 ± .46	291.77 ± 41.79
Proteinuria (mean ± SD, mg/L)	N/A	1987.69 ± 572.46	N/A	2055.17 ± 338.14
CRP (mean ± SD, mg/L)	N/A	24.02 ± 6.71	N/A	13.45 ± 2.24
IgG (mean ± SD, g/L)	N/A	12.47 ± 1.15	N/A	13.30 ± .88
C3 (mean ± SD, g/L)	N/A	.75 ± .07	N/A	.69 ± .04
C4 (mean ± SD, g/L)	N/A	.14 ± .02	N/A	.12 ± .01
Anti‐β2‐GP I (mean ± SD, RU/mL)	N/A	4.73 ± 1.07	N/A	8.50 ± 3.99
ANuA (positive, percentage)	N/A	7 (29.2%)	N/A	19 (26.0%)
ARPA (positive, percentage)	N/A	8 (33.3%)	N/A	11 (15.1%)
AHA (positive, percentage)	N/A	8 (33.3%)	N/A	26 (35.6%)
ASMA (positive, percentage)	N/A	5 (20.8%)	N/A	15 (20.5%)
SLEDAI‐2K (mean ± SD)	N/A	11.21 ± .75	N/A	9.49 ± .60

Abbreviations: AHA, anti‐dsDNA, anti‐double stranded DNA antibody; anti‐histone antibody, Anti‐β2‐GP I, anti‐beta 2 glycoprotein 1 antibody, ANuA, anti‐nuclear antibody; ARPA, anti‐ribosomal p protein antibody; ASMA, anti‐Smith antibody; C3, complement C3; C4, complement C4; CRP, C‐reactive protein; eGFR, glomerular filtration rate; HC, healthy control; HCT, haematocrit; HGB, haemoglobin; IgG, immunoglobulin G; LN, lupus nephritis; Ly, lymphocyte; Mo, monocyte; N/A, not available; Ne, neutrophils; PLT, platelet; RBC, red blood cell; SD, standard deviation; SLEDAI‐2K, Systemic Lupus Erythematosus Disease Activity Index 2000; WBC, white blood cell.

**FIGURE 1:**
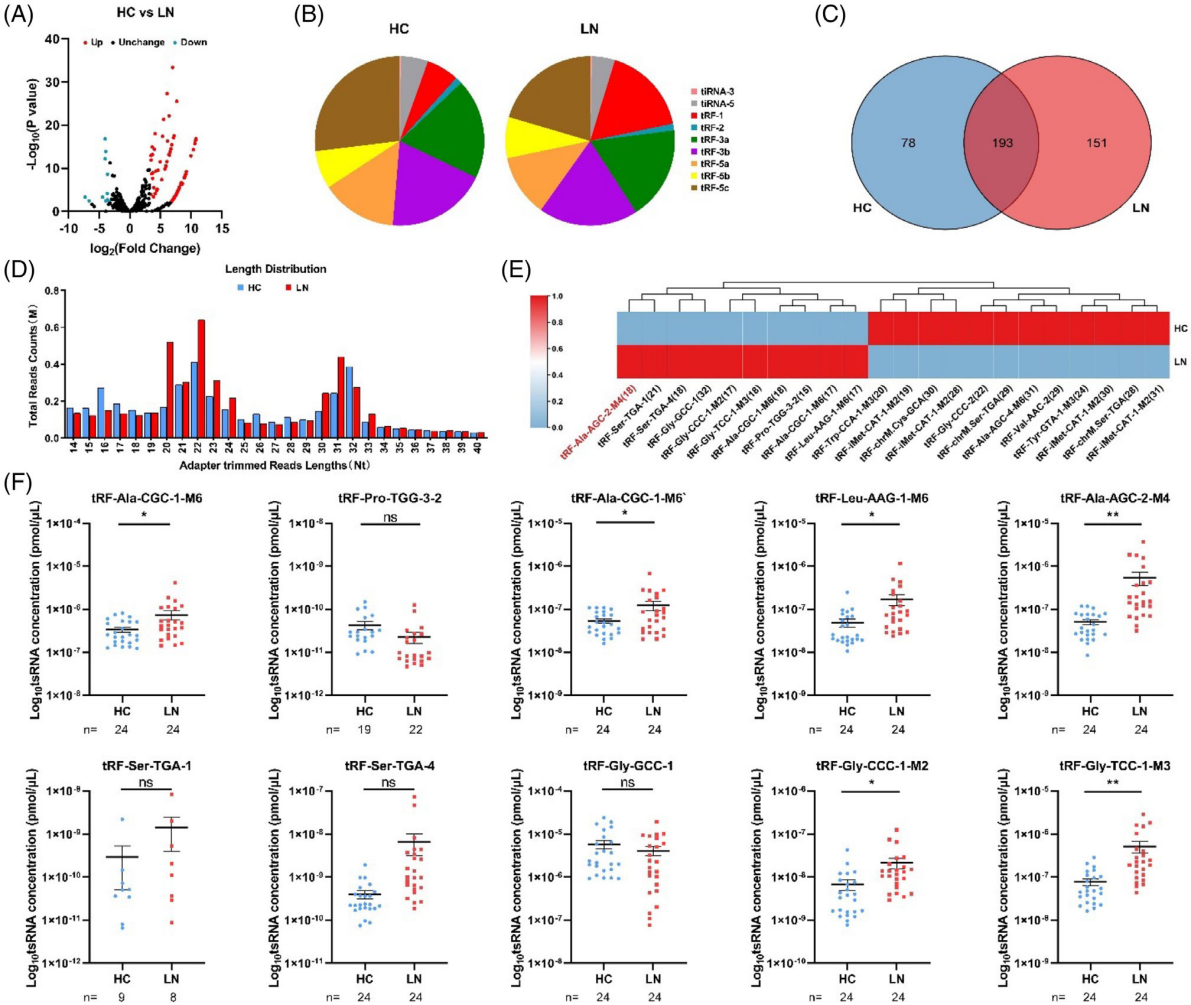
Identification of serum differential expression of tsRNA between patients with lupus nephritis (LN) and healthy controls (HC). (A) Volcano plot of differentially expressed tsRNAs. The tsRNAs represented by red (upregulation) or blue dots (downregulation) illustrated a fold change greater than 10 and a *P* value less than .01 between the two compared groups, and black dots represented tsRNAs with no differential expression. (B) Species distribution profiles of tsRNAs between LN patients and healthy controls. (C) Venn distribution of tsRNAs in LN groups and healthy controls. (D) Length distribution profiles of tsRNAs between LN patients and healthy controls. (E) Hierarchical clustering indicates the differences in tsRNA expression profiles between the two groups. (F) RT–qPCR verification of 10 differentially expressed tsRNAs in the serum of the LN group and HC group. Statistical significance was determined by unpaired two‐tailed *t* test (*P < .05, **P < .01, ns: no significant difference)

tRF‐His‐GTG‐1 has elevated levels in SLE patients but not in LN patients; hence, it can be used to distinguish LN patients from SLE patients.^8^ In this work, the total number of reads per million for tRF‐Ala‐AGC‐2‐M4 in the serum of LN patients was higher than that of HC patients (Table S2); RT–qPCR confirmed that tRF‐Ala‐AGC‐2‐M4 was exuberantly expressed in LN patients than in HC patients (*P *< .01), with area under the curve (AUC) = .7558 (validation set, Figure 2A). However, tRF‐Gly‐TCC‐1‐M3 expression was not significantly different between HC and LN (Figure S2). For LN clinical diagnosis, renal biopsy is the gold standard but is not suitable for dynamic monitoring.^9^ Anti‐dsDNA and 24‐hour proteinuria commonly used indicators for the diagnosis of LN in the clinic have low sensitivities and are time‐dependent.^2^ Further clinical analysis showed that tRF‐Ala‐AGC‐2‐M4 had a higher sensitivity (81.08 and 91.67, respectively) and AUC (.7045 and .7500, respectively) in the diagnosis of LN with negative anti‐dsDNA or 24‐hour proteinuria (Figure 2B,C; and Table S4). Surprisingly, when anti‐dsDNA or 24‐hour proteinuria was combined with tRF‐Ala‐AGC‐2‐M4, the AUC of the two for LN detection reached.8502 and .9178, respectively (Figure 2D,E; and Table S5). Correlation analysis showed that tRF‐Ala‐AGC‐2‐M4 had a strong positive correlation with C‐reactive protein (CRP) and the Systemic Lupus Erythematosus Disease Activity Index 2000 (SLEDAI‐2K), with correlation coefficient values of .3299 and .3270, respectively (Figure 2F). However, tRF‐Ala‐AGC‐2‐M4 has little correlation with other indicators (Figure S3A). According to the SLEDAI‐2K, tRF‐Ala‐AGC‐2‐M4 was highly expressed in the severely active lupus group and positively correlated with the degree of lupus activity (Figure 2G; and Table S6). However, other indicators were not significantly different among the four LN groups (Figure S3B). Thus, high levels of tRF‐Ala‐AGC‐2‐M4 in the serum correlate with the degree of activity in LN and can be an indicator for clinical evaluation for LN. Furthermore, on RT–qPCR validation of HC and four other autoimmune diseases, we verified the specificity of tRF‐Ala‐AGC‐2‐M4 in LN (Figure 2H; and Table S7). These results indicate that serum tRF‐Ala‐AGC‐2‐M4 has a certain clinical discrimination ability and can be used as a potential diagnostic marker for LN.

**FIGURE 2:**
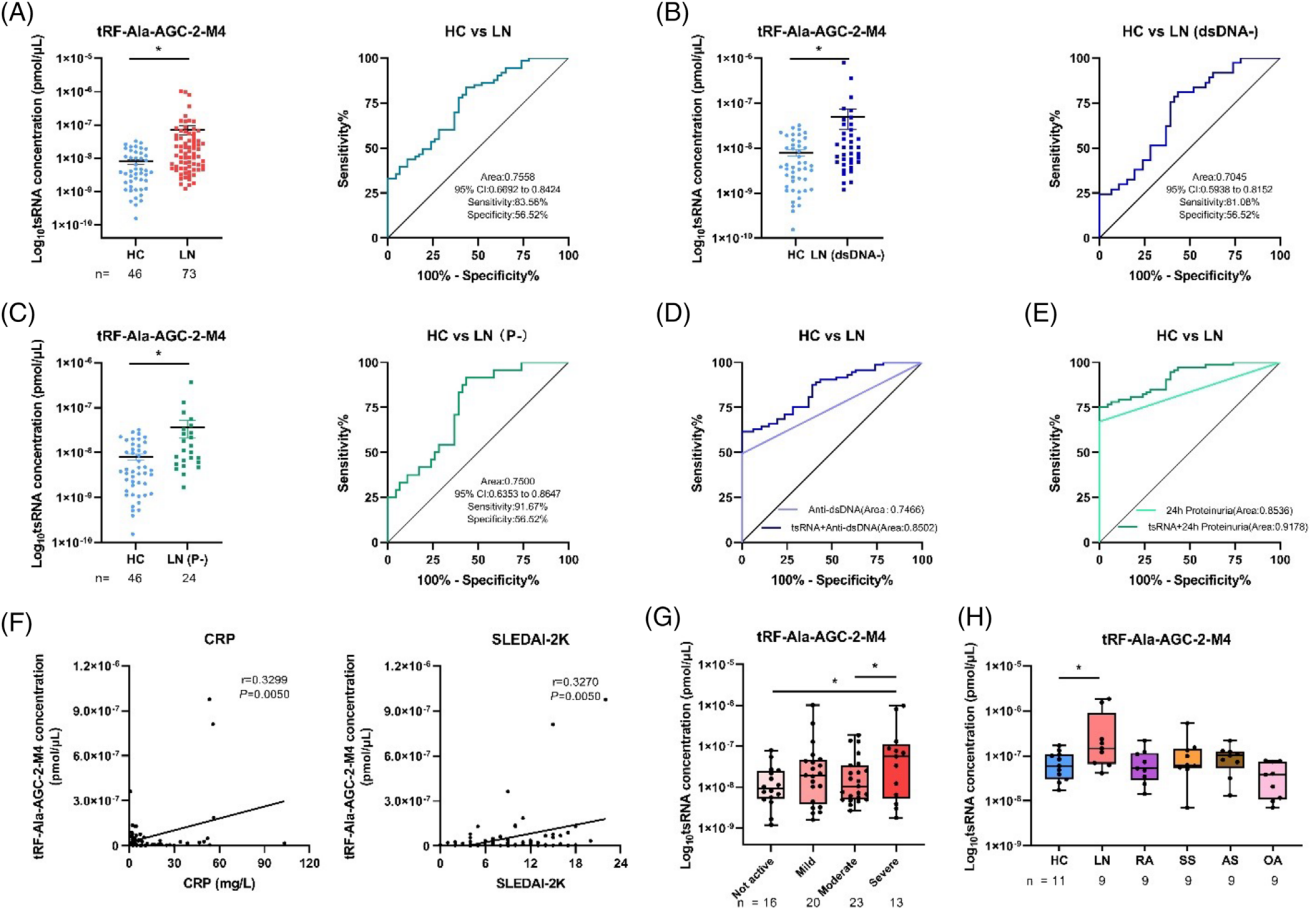
Diagnostic value of tRF‐Ala‐AGC‐2‐M4 in clinical applications. (A) Diagnostic value of tRF‐Ala‐AGC‐2‐M4 in the LN group and HC group. (B) Diagnostic value of tRF‐Ala‐AGC‐2‐M4 in anti‐dsDNA negative (dsDNA‐) LN disease individuals and the HC group. (C) Diagnostic value of tRF‐Ala‐AGC‐2‐M4 in 24‐hour proteinuria–negative (P‐) LN disease individuals and the HC group. (D) ROC combined diagnostic analysis of tRF‐Ala‐AGC‐2‐M4 and anti‐dsDNA in the LN group and HC group. (E) ROC combined diagnostic analysis of tRF‐Ala‐AGC‐2‐M4 and 24‐hour proteinuria in the LN group and healthy controls. (F) Analysis of the correlation between tRF‐Ala‐AGC‐2‐M4 and CRP and SLEDAI‐2K. (G) Differential analysis of tRF‐Ala‐AGC‐2‐M4 in different LN severity groups. (H) Differential expression analysis of tRF‐Ala‐AGC‐2‐M4 in different autoimmune diseases Statistical significance was determined by unpaired two‐tailed *t* test (*P < 0.05). SPSS binary logistic regression was used to predict the probability of joint diagnosis. Statistical correlation was determined by linear regression and Pearson correlation. AS, ankylosing spondylitis; HC, healthy control; LN, lupus nephritis; OA, osteoarthritis; RA, rheumatoid arthritis; ROC, receiver operating characteristics; SS, Sjogren's syndrome

Structural prediction showed that tRF‐Ala‐AGC‐2‐M4 originates after cleavage at the T‐loop of tRNA‐Ala‐AGC‐1, which is of the tRF‐3a type (Figure 3A). Meanwhile, the coexpression network of 123 highly expressed target genes of tRF‐Ala‐AGC‐2‐M4 was mapped (Figure 3B). Gene Ontology (GO) analysis showed that biological, cellular and molecular functions were enriched in “Response to endoplasmic reticulum stress” (GO0034976), “Focal adhesion” (GO0005925) and “Cell adhesion molecule binding” (GO0050839), respectively (Figure 3C). The LN patient body is in a state of “tension,” while tsRNAs also have a stress‐induced production pathway. Kyoto Encyclopedia of Genes and Genomes (KEGG) probing confirmed the pathway assignment in the “Huntington disease” (hsa05016) signalling pathway. More importantly, the wiki pathway was enriched in the “Nephrotic syndrome” (WP4758) signalling pathway. Meanwhile, these 10 highly expressed tsRNAs in LN serum were enriched in the above pathway (Figure S4). LN is a form of glomerulonephritis, and massive autoantibody involvement of the kidney induces LN, which is closely related to secondary nephrotic syndrome.^10^ Bioinformatics tools validated that tRF‐Ala‐AGC‐2‐M4 can be crucial for the development of LN.

**FIGURE 3:**
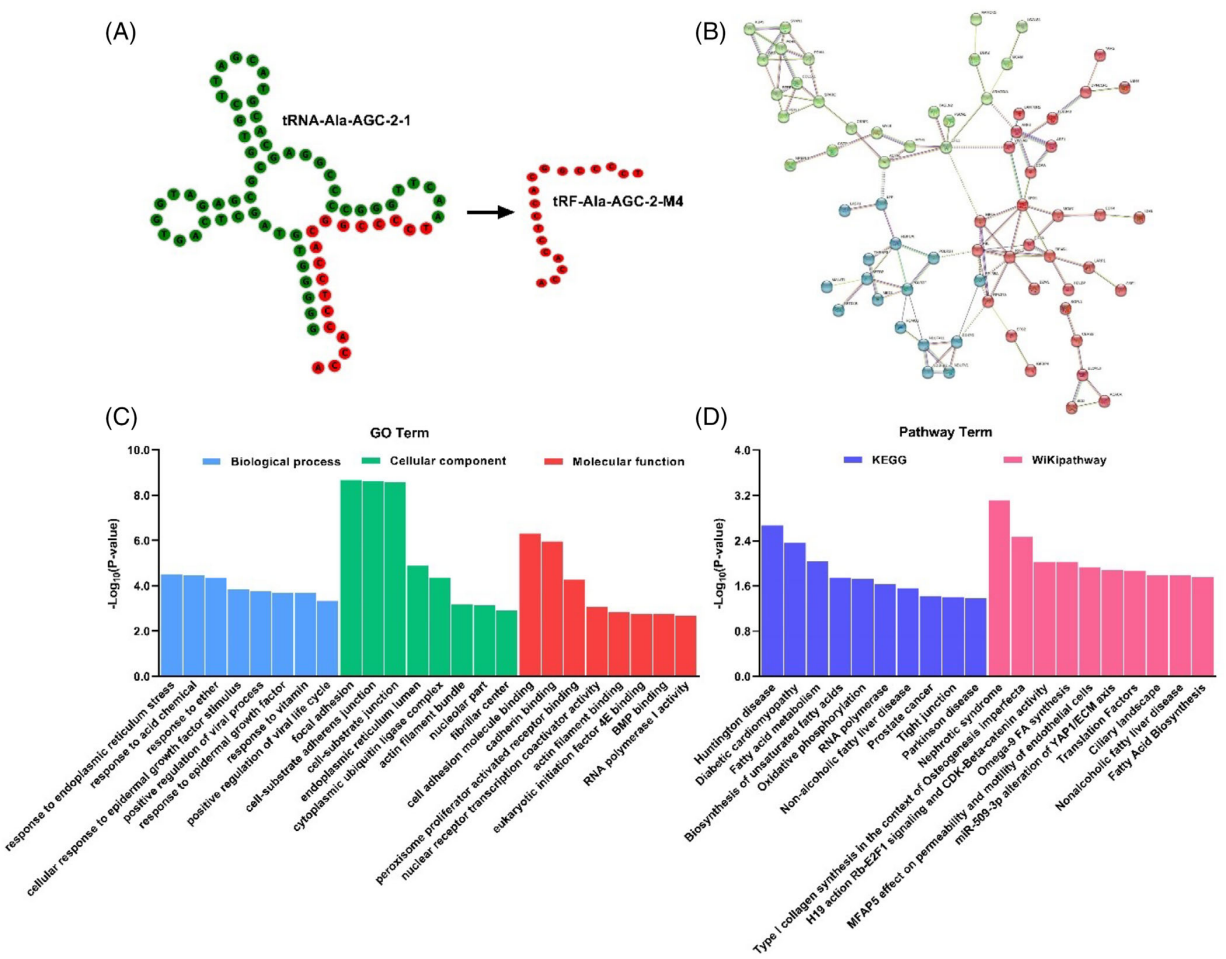
The biological functions of tRF‐Ala‐AGC‐2‐M4. (A) Secondary structure prediction of tRF‐Ala‐AGC‐2‐M4. (B) Network interaction mapping of tRF‐Ala‐AGC‐2‐M4 with genes where the minimum required interaction score was set to .5 and k‐means clustering was adopted. (C, D) GO terms and signal pathway analysis of tRF‐Ala‐AGC‐2‐M4. The conditions of cluster analysis were as follows: tsRNAs were highly expressed and coexpressed only in tRF‐gene interactions (TGIs), while the number of genes cotargeted by tsRNAs was at least 1. Bioinformatics analysis and structure prediction of tRF‐Ala‐AGC‐2‐M4 was performed via the tRNAdb database (http://trna.bioinf.uni‐leipzig.de/), network map prediction via STRING (https://cn.string‐db.org/) and GO and pathway analysis via tRFTar (http://www.rnanut.net/tRFTar/)

In summary, our study is the first to report the superior diagnostic ability of tRF‐Ala‐AGC‐2‐M4 and confirms that tsRNA markers are specifically highly expressed in LN serum and validated by sequencing and RT–qPCR of serum tsRNAs from patients with LN. Further clinical evaluation showed that tRF‐Ala‐AGC‐2‐M4 has good diagnostic value for LN and is closely related to the severity of LN. Finally, bioinformatics tools also predicted the involvement of tRF‐Ala‐AGC‐2‐M4 in the nephrotic syndrome signalling pathway. These results may provide new insights for accomplishing the goal of early diagnosis of LN and can be of value for making treatment decisions.

## FUNDING INFORMATION

This study has been supported by the National Natural Science Foundation of China (No. 61971216) and the Key Research and Development Project of Jiangsu Province (No. BE2019603, BE2020768).

## CONFLICT OF INTEREST

All authors affirm no conflict of interest.

## Supporting information



Supplementary information
