# Supplementary material for: Serum tsRNA as a novel molecular diagnostic biomarker for lupus nephritis
Source: Clin Transl Med. 2022 May 20;12(5):e830. doi: 10.1002/ctm2.830 (PMC9121311; doi:10.1002/ctm2.830)
Supplement: Supplementary file 1 — Supplementary information [file CTM2-12-e830-s001.docx]

**Supplementary materials**

**Materials** **and** **methods**

***Experimental design and sample collection***

The study was divided into three stages: sequencing, training, and verification. Moreover, the value of the selected biomarkers was determined through clinical evaluation and functional analysis. Serum samples from 93 healthy individuals (HC) and 130 lupus nephritis patients (LN) were collected from Drum Tower Hospital affiliated with Nanjing University School of medicine, informed consent was priroly obtained for inclusion in the study (**Table 1**). Initially, total RNA enriched form 23 HC serum and 33 LN serum were selected for tsRNAs sequencing. In the later stage, 24 HC and 24 LN serum were randomly selected for the screening of differentially expressed tsRNAs. Finally, a large cohort consisting 46 HC and 73 LN serum were used for the validation of candidate tsRNAs. All the patients enrolled in the study fulfilled the revised criteria for SLE established by the 1997 American College of Rheumatology (ACR). SLE disease activity was recorded by Systemic Lupus Erythematosus Disease Activity Index 2000 (SLEDAI) score at the time of blood being drawn. Classification criteria: scores between 1-4 indicates basic inactivity, 5-8 mild activity, 9-14 moderate activity, and ≥ 15 indicate severe activity. This study was approved by the ethics committee of Drum Tower hospital affiliated with the Medical College of Nanjing University (ID: 2020-327-01) and conducted in accordance with the principle set forth under the 1989 Declaration of Helsinki. Written consent was obtained from all the subjects included in the study.

***Small RNA sequencing***

In order to investigate the expression pattern of tsRNAs in HC and LN serum, we performed small RNA sequencing utilizing total serum RNA. To enrich the total serum RNA we utilized Trizol reagent (Invitrogen). The concentration of enriched RNA was quantified using Qubit.4.0 (Life technologies), a fluorescence quantifier. Since tsRNAs contain abundant modifications derived from tRNA, a prominent interfering factor for efficient construction of sequencing libraries. To deal with it total RNA samples were subjected to the following treatments before library preparation: 3'-aminoacyl (charged) deacylation to 3'-OH for 3'-adapter ligation, 3'-CP (2', 3'-cyclic phosphate) removal to 3'-OH for 3'-adapter ligation, 5'-OH (hydroxyl) phosphorylation to 5'-P for 5'-adapter ligation, deacylation of m1A and m3C methylation to enable efficient reverse transcription. The integrity of RNA samples was established by performing Agarose gel electrophoresis. The completed libraries were quantified using the Agilent 2100 Bioanalyzer. The single stranded DNA obtained from double stranded DNAs in the library was treated with 0.1 M NaOH for sequencing. The single stranded DNA with a concentration of 1.8 pM was loaded onto the NextSeq 500/550 V2 kit (#FC-404-2005, Illumina), and the sequencing operation was performed in the NextSeq system for 50 cycles according to the instructions.

***RNA extraction and RT-qPCR assays***

Total RNA extracted from 100 μL serum was dissolved in 20 μL DEPC water as previously described. For assessing the concentration and purity of total RNA we used onedrop-2000 (nanodrop technologies). TaqMan probe-based RT-qPCR assays were performed using a commercial kit (vazyme biotech). Briefly, 2 μL of total RNA was first mixed with 5 × gDNA wiper mix and incubated at 42 °C for 2 min to remove contamination of genomic DNA. Incubate the mixture at 25 °C for 5 min, 50 °C for 15 min, and 85 °C for 5 min. Finally, used RT-qPCR on a BIO-RAD CFX96 Deep Well DX system (Bio-Rad) with a TaqMan custom synthesized tsRNA probe (GenScript Biotech Corp) as instructed (**Table S1**). PCR program for tsRNAs included the following steps: 95 °C for 10 min, followed by 40 cycles at 95 °C for 15 s and 60 °C for 1 min. The threshold cycle (CT) values were determined using a fixed threshold setting.

***tsRNA bioinformatics analysis***

The tsRNA produced by tRNA fragmentation participates in the biological process of the organism and the regulation of various signal pathways. We use tsRFun (http://rna.sysu.edu.cn/tsRFun/links.php) to predict the secondary structure of tsRNAs, and use string (https://www.string-db.org) to analyze the target tsRNA target diagram of the gene interaction network. Finally, tRFTar (http://www.rnanut.net/tRFTar/) was used to perform gene ontology and pathway enrichment analysis for the selected tsRNA.

***Statistical* *Analysis***

All statistical data were expressed as mean ± standard deviation (mean ± SD), and categorical data were described using frequencies or percentages. Differences between two groups were evaluated by student's t-test, and one-way analysis of variance (ANOVA) was used for comparison among multiple groups (n ≥ 3). Receiver operating characteristic (ROC) curves and area under the curve (AUC) were used to establish the utility of serum tsRNAs as LN biomarkers. Binary logistic regression was used to predict the probability of joint diagnosis with corresponding 95% confidence intervals (CI). All descriptive statistics, prediction of joint diagnosis probability, and one-way ANOVA were performed using SPSS 24.0 (SPSS Inc., Chicago, IL, USA). Scatterplots and ROC curves were obtained by using Graphpad prism 8.0.2 (Graphpad software, Inc, San Diego, CA, USA). *P*-value ≤ 0.05 was considered statistically significant.

***Data and materials availability***

The raw data of tsRNA sequencing generated in this study can be accessed at GEO database with the accession number: GSE179950. The main findings of this study can be confirmed in the manuscript and supplementary material. However, additional data supporting the manuscript finding can be shared upon reasonable request from the corresponding authors.

**Table S1:** Mapping summary of sequencing.

| **Sample** | **Total Read** | **Mat-tRNA** | **Mat-tRNA  (%)** | **Pre-tRNA** | **Pre-tRNA  (%)** | **miRNA** | **miRNA  (%)** |
| --- | --- | --- | --- | --- | --- | --- | --- |
| **HC** | 5999363 | 224668 | 5.59 | 5892 | 0.15 | 581142 | 14.47 |
| **LN** | 6488953 | 654518 | 14.17 | 38515 | 0.83 | 1116871 | 24.18 |

**Table S2:** Sequencing information of 10 candidate tsRNAs.

| **tRF_ID** | **Sequence** | **Type** | **Length** | **HC_CPM** | **LN_CPM** |
| --- | --- | --- | --- | --- | --- |
| **tRF-Ala-CGC-1-M6** | UCCCCGGCAUCUCCACCA | tRF-3a | 18 | 117.11 | 1578.57 |
| **tRF-Pro-TGG-3-2** | UCGUGGCUACUGUUU | tRF-1 | 15 | 0.00 | 253.96 |
| **tRF-Ala-CGC-1-M6'** | CCCCGGCAUCUCCACCA | tRF-3a | 17 | 13.01 | 347.75 |
| **tRF-Leu-AAG-1-M6** | UCCCACCGCUGCCACCA | tRF-3a | 17 | 30.36 | 337.65 |
| **tRF-Ala-AGC-2-M4** | UCCCCGGCACCUCCACCA | tRF-3a | 18 | 125.78 | 949.45 |
| **tRF-Ser-TGA-1** | GAAGCGGGUGCUCUUAUUUUU | tRF-1 | 21 | 8.67 | 1517.97 |
| **tRF-Ser-TGA-4** | AUCCUGUCGGCUACGCCA | tRF-3a | 18 | 21.69 | 167.38 |
| **tRF-Gly-GCC-1** | GCAUGGGUGGUUCAGUGGUAGAAUUCUCGCCU | tRF-5c | 32 | 3747.40 | 8263.68 |
| **tRF-Gly-CCC-1-M2** | UCCCGGCCAAUGCACCA | tRF-3a | 17 | 0.00 | 36.07 |
| **tRF-Gly-TCC-1-M3** | UUCCCGGCCAACGCACCA | tRF-3a | 18 | 0.00 | 30.30 |

**Abbreviations:** tRF, tRNA-derived fragment; CPM, counts per million of total aligned reads.

**Table S3:** Specific primer information for 10 candidate tsRNAs.

| **tRF ID** | **Primer sequence** | **Tm (°C)** |
| --- | --- | --- |
| **tRF-Ala-CGC-1-M6** | RT_GTCGTATCCAGTGCAGGGTCCGAGGTATTCGCACTGGATACGACTGGTGG | 63.0 |
|  | FP_CGCGTCCCCGGCATCT |  |
| **tRF-Pro-TGG-3-2** | RT_GTCGTATCCAGTGCAGGGTCCGAGGTATTCGCACTGGATACGACAAACAG | 63.0 |
|  | FP_GCGCGCGTCGTGGCTA |  |
| **tRF-Ala-CGC-1-M6'** | PT_GTCGTATCCAGTGCAGGGTCCGAGGTATTCGCACTGGATACGACTGGTGG | 68.1 |
|  | FP_GCGCGCCCCGGCATCT |  |
| **tRF-Leu-AAG-1-M6** | PT_GTCGTATCCAGTGCAGGGTCCGAGGTATTCGCACTGGATACGACTGGTGG | 64.6 |
|  | FP_GCGCGTCCCACCGCTG |  |
| **tRF-Ala-AGC-2-M4** | PT_GTCGTATCCAGTGCAGGGTCCGAGGTATTCGCACTGGATACGACTGGTGG | 65.5 |
|  | FP_CGCGTCCCCGGCACCT |  |
| **tRF-Ser-TGA-1** | PT_GTCGTATCCAGTGCAGGGTCCGAGGTATTCGCACTGGATACGACAAAAAT | 59.0 |
|  | FP_CGGAAGCGGGTGCTCTT |  |
| **tRF-Ser-TGA-4** | PT_GTCGTATCCAGTGCAGGGTCCGAGGTATTCGCACTGGATACGACTGGCGT | 62.4 |
|  | FP_GCGCGATCCTGTCGGCT |  |
| **tRF-Gly-GCC-1** | PT_GTCGTATCCAGTGCAGGGTCCGAGGTATTCGCACTGGATACGACAGGCGA | 60.6 |
|  | FP_TGGGTGGTTCAGTGGTAGAATTC |  |
| **tRF-Gly-CCC-1-M2** | PT_GTCGTATCCAGTGCAGGGTCCGAGGTATTCGCACTGGATACGACTGGTGC | 65.5 |
|  | FP_GCGCGTCCCGGCCAAT |  |
| **tRF-Gly-TCC-1-M3** | PT_GTCGTATCCAGTGCAGGGTCCGAGGTATTCGCACTGGATACGACTGGTGC | 63.5 |
|  | FP_CGCGTTCCCGGCCAAC |  |
| **Universal mQ Primer R** | FR_AGTGCAGGGTCCGAGGTATT |  |

**Abbreviations:** tRF, tRNA-derived fragment; Tm, melting temperature.

**Table S4:** Comparison of tRF-Ala-AGC-2-M4 with anti-dsDNA and 24-hour proteinuria in the diagnosis of LN

| **tRF-Ala-AGC-2-M4** | **24-hour Proteinuria** | | **Anti-dsDNA** | | **Total** |
| --- | --- | --- | --- | --- | --- |
|  | **Negitive (-)** | **Positive (+)** | **Negitive (-)** | **Positive (+)** |  |
| **Negitive (-)** | 2 | 10 | 7 | 5 | 12 |
| **Positive (+)** | 22 | 39 | 30 | 31 | 61 |
| **Total** | 24 | 49 | 37 | 36 | 73 |

**Abbreviations:** tRF, tRNA-derived fragment; Anti-dsDNA, anti-double stranded DNA antibody.

**Table S5:** Detailed data of ROC curve.

| **Marker** | **Group** | **95% CI** | **Sensitivity (%)** | **Specificity (%)** | **P-value** | **AUC** |
| --- | --- | --- | --- | --- | --- | --- |
| **tRF-Ala-AGC-2-M4** | LN vs HC | 0.6692 to 0.8424 | 83.56 | 56.52 | <0.0001 | 0.7558 |
| **tRF-Gly-TCC-1-M3** | LN vs HC | 0.4137 to 0.7266 | 86.67 | 29.17 | 0.3794 | 0.5701 |
| **tRF-Ala-AGC-2-M4** | LN (P-) vs HC | 0.6353 to 0.8647 | 91.67 | 56.52 | 0.0006 | 0.7500 |
| **tRF-Ala-AGC-2-M4** | LN (dsDNA-) vs HC | 0.5938 to 0.8152 | 81.08 | 56.52 | 0.0014 | 0.7045 |
| **proteinuria** | LN vs HC | 0.7639 to 0.9074 | 67.12 | 100.00 | <0.0001 | 0.8356 |
| **anti-dsDNA** | LN vs HC | 0.6607 to 0.8324 | 49.32 | 100.00 | <0.0001 | 0.7466 |
| **tRF-Ala-AGC-2-M4 + proteinuria** | LN vs HC | 0.8713 to 0.9644 | 75.34 | 100.00 | <0.0001 | 0.9178 |
| **tRF-Ala-AGC-2-M4 + anti-dsDNA** | LN vs HC | 0.7845 to 0.9159 | 61.64 | 100.00 | <0.0001 | 0.8502 |

**Abbreviations:** LN, lupus nephritis; HC, healthy control; tRF, tRNA-derived fragment; 95% CI: 95% confidence interval; AUC, Area Under Curve; P-, 24-hour Proteinuria Negitive; dsDNA-, anti-dsDNA Negitive; Anti-dsDNA, anti-double stranded DNA antibody.

**Table S6:** Clinical information of patients with different severity of LN.

| **Index** | **Not activity**  **(n=16)** | **Mild**  **(n=20)** | **Moderate**  **(n=23)** | **Severe**  **(n=13)** |
| --- | --- | --- | --- | --- |
| **Sex (Female, percentage)** | 16 (100.00) | 19 (95.00%) | 20 (86.96%) | 12 (92.31%) |
| **Age (year)** | 42.56 ± 14.50 | 43.35 ± 14.12 | 43.91 ± 14.00 | 36.54 ± 12.86 |
| **Proteinuria (mg/L)** | 28.57 ± 181.45^b^ | 1645.73 ± 2452.98^ab^ | 2812.62 ± 3328.20^a^ | 2799.59 ± 2572.22^a^ |
| **eGFR** | 132.59 ± 64.382 | 86.30 ± 55.93 | 112.20 ± 49.16 | 120.39 ± 56.51 |
| **CRP (mg/L)** | 20.54 ± 27.71 | 11.76 ± 15.06 | 10.61 ± 14.36 | 12.36 ± 19.83 |
| **IgG (g/L)** | 14.23 ± 3.69 | 13.68 ± 8.96 | 12.93 ± 8.03 | 12.33 ± 5.70 |
| **C3 (g/L)** | 0.98 ± 0.31^a^ | 0.70 ± 0.34^ab^ | 0.59 ± 0.33^b^ | 0.47 ± 0.21^b^ |
| **C4 (g/L)** | 0.17 ± 0.08^a^ | 0.13 ± 0.09^ab^ | 0.11 ± 0.09^ab^ | 0.08 ± 0.08^b^ |
| **Anti-dsDNA (IU/L)** | 177.54 ± 184.98 | 316.18 ± 371.18 | 285.93 ± 327.57 | 401.18 ± 423.76 |
| **Anti-β2-GP I (RU/mL)** | 5.7 ± 2.91 | 3.22 ± 4.19 | 17.02 ± 47.45 | 2.91 ± 2.59 |
| **SLEDAI-2K** | 2.63 ± 1.63^d^ | 7.40 ± 1.57^c^ | 11.96 ± 1.46^b^ | 16.85 ± 2.19^a^ |

**Abbreviations:** LSD method in ANOVA was used to compare the mean values of multiple groups. a and b represent differences in the mean values between groups. eGFR, glomerular filtration rate; CRP, C-reactive protein; IgG, immunoglobulin G; C3,Complement C3;C4, Complement C4; anti-dsDNA, anti-double stranded DNA antibody; Anti-β2-GP I , SLEDAI, systemic lupus erythematosus disease activity index

**Table S7:** Statistics of clinical information for patients with lupus nephritis (LN), rheumatoid arthritis (RA), Sjogren's syndrome (SS), ankylosing spondylitis (AS) and osteoarthritis (OA).

| **Index** | **Healthy contral**  **(HC, n=11)** | **Lupus nephritis**  **(LN, n=9)** | **Rheumatoid Arthritis**  **(RA, n=9)** | **Sjogrensyndrome (SS, n=9)** | **Ankylosing spondylitis (AS, n=9)** | **Osteoarthritis (OA, n=9)** |
| --- | --- | --- | --- | --- | --- | --- |
| **Sex (Female, percentage)** | 11 (100.00%) | 8 (88.89%) | 8 (88.89%) | 8 (88.89%) | 4 (44.44%) | 9 (100.00%) |
| **Age (Mean ± SD, year)** | 27.27 ± 3.20 | 34.78 ± 11.77 | 56.44 ± 12.27 | 62.44 ± 13.13 | 32.33 ± 12.53 | 65.11 ± 6.88 |
| **C3 (Mean ± SD, g/L)** | N/A | 0.81 ± 0.22 | N/A | 1.05 ± 0.33 | N/A | N/A |
| **C4 (Mean ± SD, g/L)** | N/A | 0.14 ± 0.06 | N/A | 0.20 ± 0.08 | N/A | N/A |
| **IgG (Mean ± SD, g/L)** | N/A | 10.38 ± 2.69 | N/A | 13.87 ± 5.92 | N/A | N/A |
| **CRP (Mean ± SD, g/L)** | N/A | 4.58 ± 4.21 | 4.46 ± 2.99 | 6.88 ± 4.71 | 22.34 ± 21.99 | 14.41 ± 13.20 |
| **eGFR (Mean ± SD)** | 137.38 ± 22.39 | 116.63 ± 57.56 | 148.04 ± 40.37 | 109.30 ± 17.39 | N/A | 120.24 ± 20.73 |

**Abbreviations:** C3,Complement C3;C4, Complement C4; IgG, immunoglobulin G; CRP, C-reactive protein; eGFR, glomerular filtration rate; N/A, not available.

**
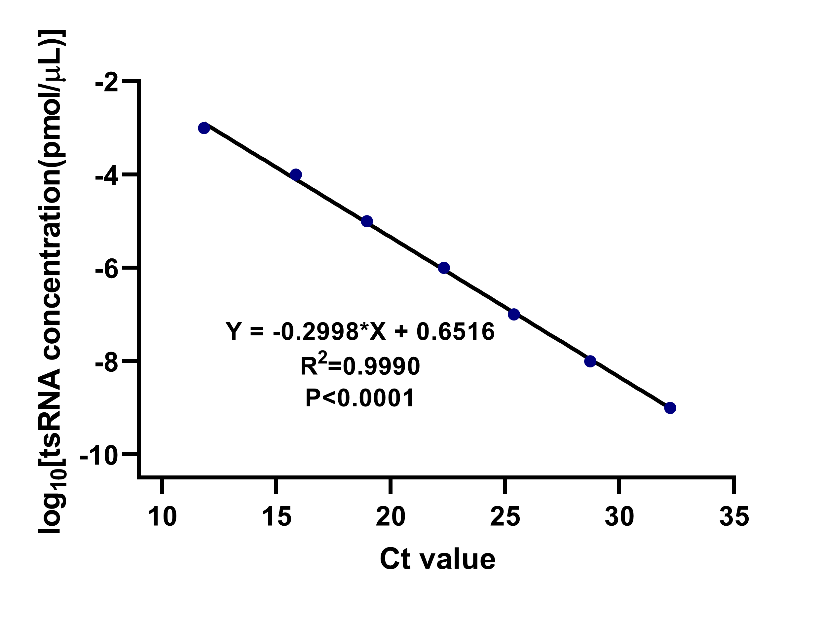
**

**Figure S1:** Standard curves of tsRNA concentrations and CT values. Statistical correlation was determined by linear regression and pearson correlation.

**
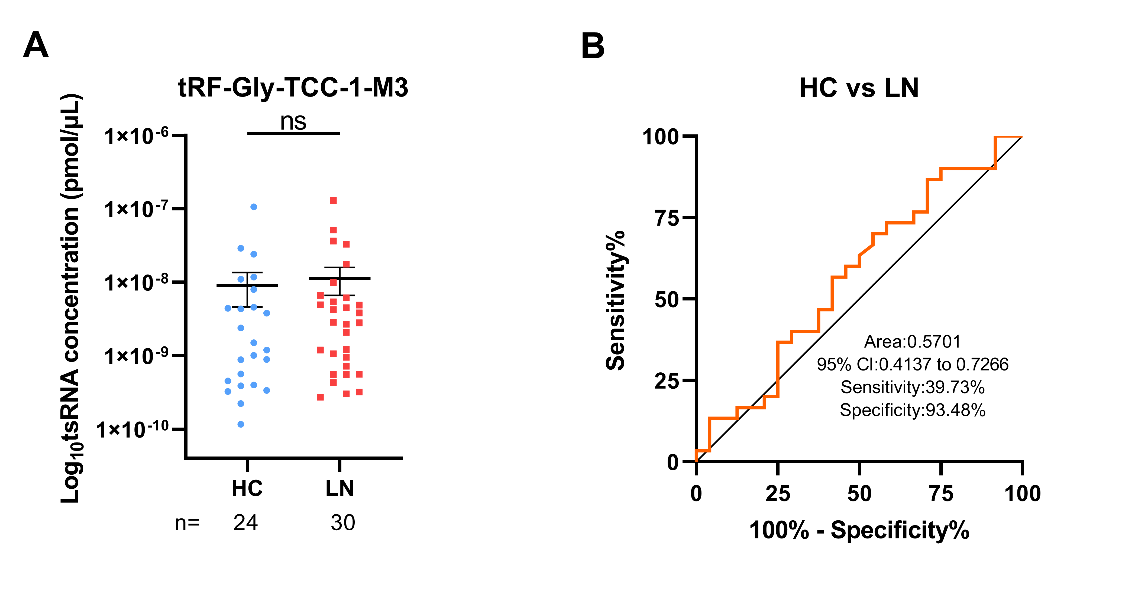
**

**Figure S2:** **Diagnostic use of serum tRF-Gly-TCC-1-M3 in LN.** **(A)** RT-qPCR verification of tRF-Gly-AGC-2-M4 in LN group and HC group. **(B)** ROC analysis of tRF-Ala-AGC-2-M4 in LN group and HC group. Statistical significance was determined by unpaired two-tailed t-test (ns: No significant difference).


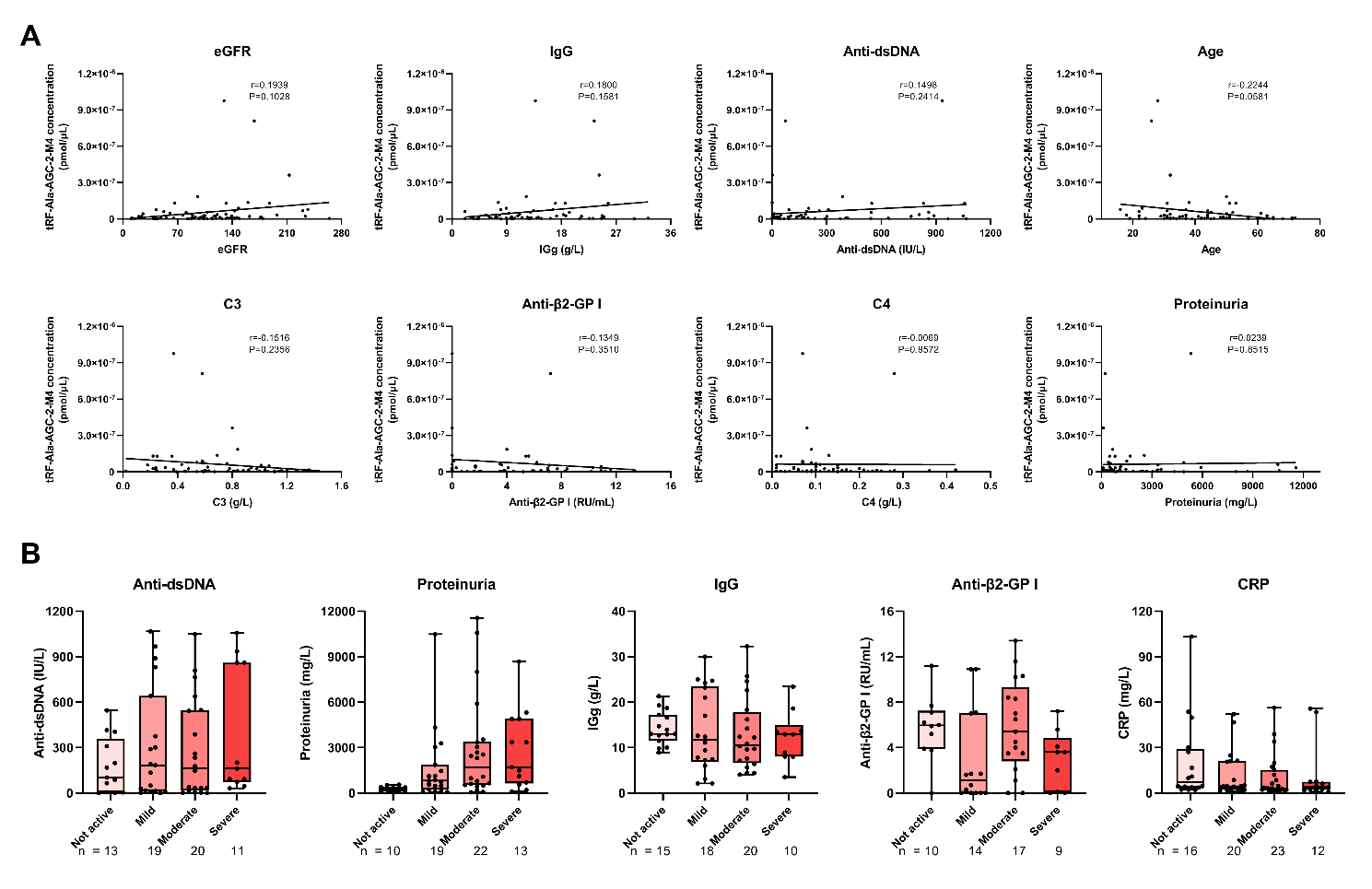


**Figure S3:** **(A)** Correlation analysis between tRF-Ala-AGC-2-M4 and clinical test indicators. **(B)** Differential analysis of Anti-dsDNA, proteinuria, IgG, anti-β2-GP I and CRP in different severity of LN group. Statistical correlation was determined by linear regression and pearson correlation.


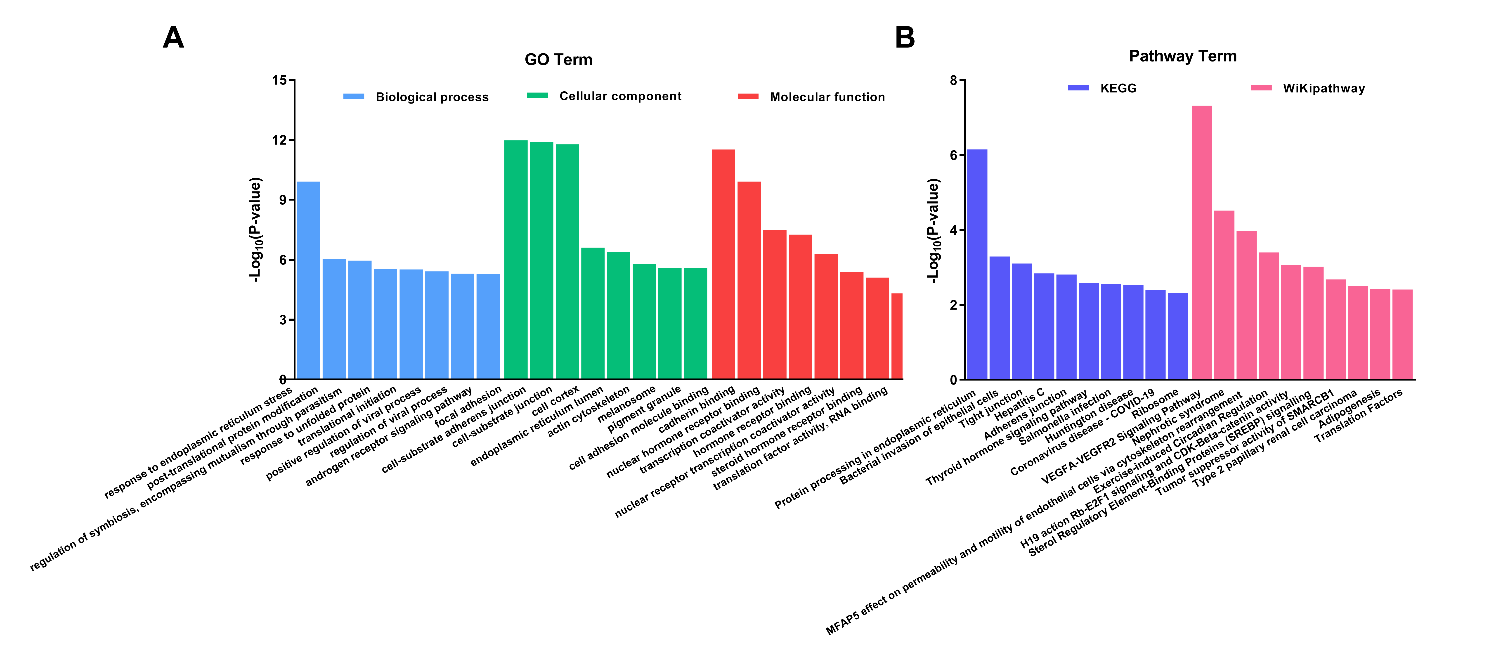


**Figure S4:** **(A, B)** GO and Pathway enrichment analysis of 10 highly expressed tsRNAs.


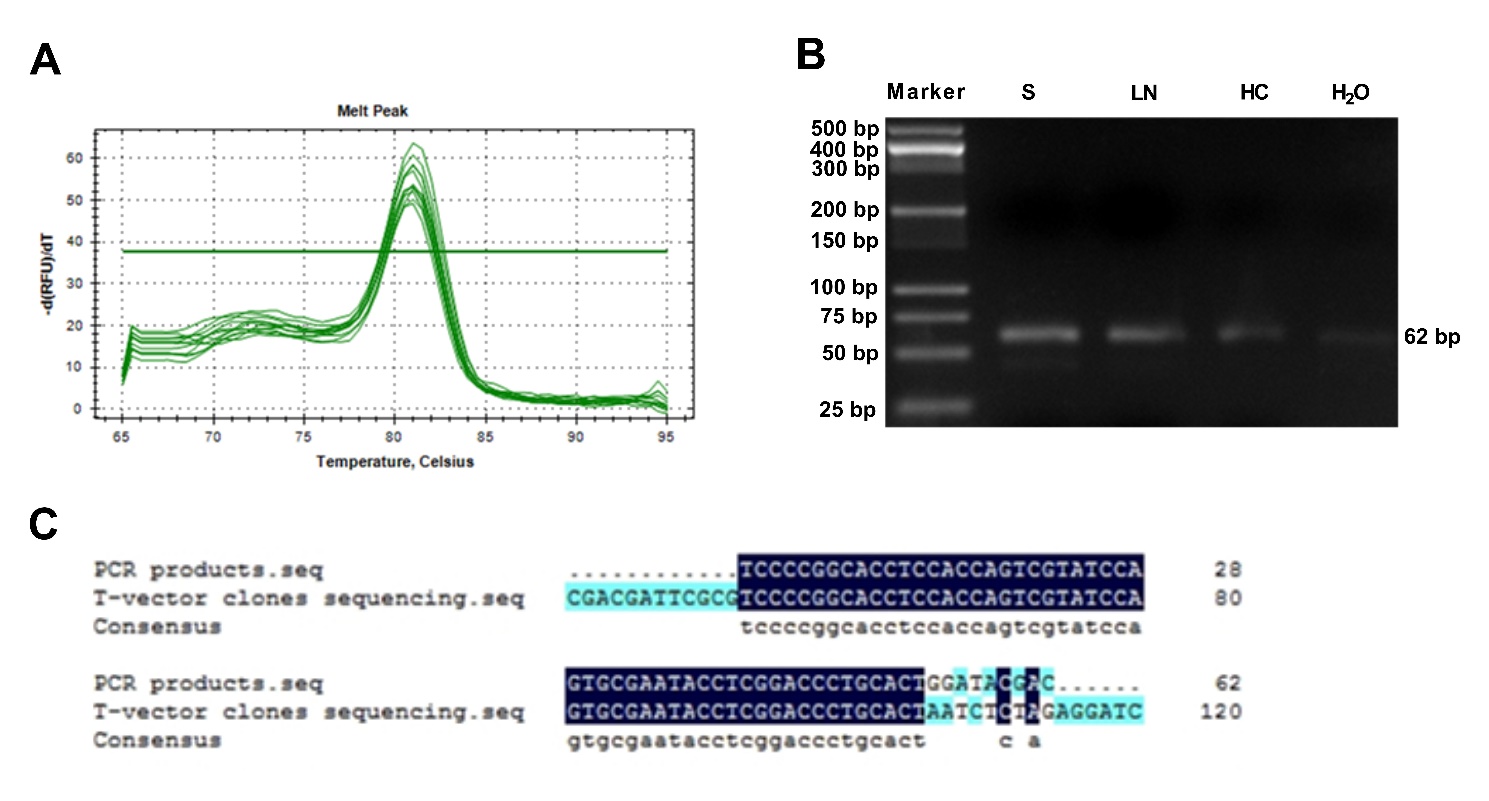


**Figure S5:** Validation of specificity of tRF-Ala-AGC-2-M4 amplification by stem-loop RT-qPCR. **(A)** A single melting peak profile of tRF-Ala-AGC-2-M4 amplified by stem-loop RT-qPCR. **(B)** Agarose gel electropherogram of RT-qPCR product of tRF-Ala-AGC-2-M4 (band size: 62 bp). S: Artificially synthesized tRF-Ala-AGC-2-M4 amplification product; LN: LN serum RNA amplification product; HC: HC serum RNA amplification product; H_2_O: H_2_O amplification products. **(C)** Sequence alignment of PCR products with sequencing results of T-vector clones. Black region: the sequence of the PCR product coincides part of the sequence sequenced by the T-vector clone.
